# The Radioprotective Effect of LBP on Neurogenesis and Cognition after Acute Radiation Exposure

**DOI:** 10.2174/0118744710274008231220055033

**Published:** 2024-01-09

**Authors:** Gang Yin, Qinqi Wang, Tongtong Lv, Yifan Liu, Xiaochun Peng, Xianqin Zeng, Jiangrong Huang

**Affiliations:** 1 Department of Neurology, Jingzhou Hospital Affiliated to Yangtze University, Jingzhou, Hubei, China;; 2 Department of Internal Medicine, Wuhan No.1 Hospital, Wuhan, Hubei, China;; 3 Department of Pathophysiology, School of Basic Medicine, Health Science Center, Yangtze University, Jingzhou, Hubei, China;; 4 Department of Gynaecology and Obstetrics, Tongji Medical College, Union Hospital, Huazhong University of Science and Technology, Wuhan, Hubei, China;; 5 Department of Integrative Medicine, Health Science Center, Yangtze University, Jingzhou, Hubei, China

**Keywords:** LBP, radiation exposure, radioprotective, neurogenesis, oxidative stress, brain damage, cognitive impairment

## Abstract

**Background:**

Radiation exposure has been linked to the development of brain damage and cognitive impairment, but the protective effect and mechanism of Lycium barbarum pills (LBP) on radiation-induced neurological damage remains to be clarified.

**Methods:**

Behavioral tests and immunohistochemical studies were conducted to evaluate the protective effects of LBP extract (10 g/kg orally daily for 4 weeks) against radiation-induced damage on neurogenesis and cognitive function in Balb/c mice exposed to 5.5 Gy X-ray acute radiation.

**Results:**

The results showed that the LBP extract significantly improved body weight loss, locomotor activity and spatial learning and memory. Immunohistochemical tests revealed that the LBP extract prevented the loss of proliferating cells, newly generated neurons and interneurons, especially in the subgranular area of the dentate gyrus.

**Conclusion:**

The findings suggest that LBP is a potential neuroprotective drug for mitigating radiation-induced neuropsychological disorders.

## 1 INTRODUCTION

To our knowledge, radiation is widespread in nature and affects people's health anytime and anywhere [1-3]. Sustained exposure to low doses of radiation may exert significant adverse effects on the human body [4-6]. As the utilization of X-ray computed tomography in medical diagnostics and radiotherapy continues to rise, the accumulation of nuclear waste in hospitals has increased significantly. This has become the primary source of anthropogenic radiation exposure in the general population, contributing to approximately 14% of the total annual radiation exposure from all sources globally [7-9]. Since the head region requires optimal diagnostic high-resolution methods based primarily on ionizing radiation (IR), studies have demonstrated that low doses of IR can not only induce cancer but also neurodegenerative diseases such as Parkinson's or Alzheimer's disease [8-10].

Radiation brain injury is a continuous and dynamic process [11-13], which is generally divided into three stages: (1) Acute reaction, which occurs within 2 weeks after the initial exposure to radiation, and patients may experience nausea, vomiting, headache, fatigue, neurological symptoms and increased signs and other symptoms; (2) Early delayed reaction, which occurs 2 weeks to 6 months following exposure to radiation. These complications may be linked to temporary demyelination processes triggered by the disruption of the blood-brain barrier or specific dysfunction in oligodendrocytes. This includes conditions like narcolepsy syndrome, transient cognitive impairment, and subacute rhomboid encephalitis/encephalitis; (3) Delayed reactions. Reactions that occur months to years after radiation, including focal brain necrosis and late delayed reactions with mild to moderate cognitive impairment, are irreversible and destructive and hence require special attention [14-16]. Radiation deposits energy into cellular biomolecules, which causes chemical changes including DNA damage. Radiolysis of water produces reactive oxygen species (ROS), ROS is potentially amplified by radiation-induced energy-producing mitochondrial damage, resulting in further damage to nucleic acids, proteins and lipids, and components of the extracellular matrix [17-19]. Moreover, radiation energy causes water in the extracellular environment to split and produce various forms of ROS, including superoxide anions, hydroxyl radicals and hydrogen peroxide, as well as malondialdehyde (MDA), an oxidation reaction product. These highly active entities can potentially damage normal tissues [20]. In addition, radiation can increase the production of endogenous ROS by mitochondria and change the permeability of mitochondrial membrane [21, 22].

Traditional Chinese Medicine prescription Lycium barbarum pills (LBP) comprise Lycium barbarum berry and Huangjing. Clinically, LBP is a promising neuronal protective agent that is known to alleviate oxidative stress, inflammation, cell apoptosis and cell death, with minimal side effects [23]. Lycium barbarum berry possesses antioxidant properties and hence can treat several diseases such as neurodegenerative diseases, cardiovascular disease and cancer [24]. Our previous study showed that Lycium barbarum berry may be an alternative food supplement for preventing radiation-induced neuron loss and neuropsychological disorders [25]. The main chemical components of Huangjing include steroidal saponins, polysaccharides, flavonoids, alkaloids, among other molecules, which have antioxidant activity, anti-aging activity, anti-inflammatory effect and immune-modulating properties. For this reason, it is a potential agent for the management of Alzheimer's disease and other diseases [26-28]. In this study, we evaluated the radioprotective effect of LBP on brain neurogenesis and cognition using a mouse model exposed to radiation.

## 2 MATERIALS AND METHODS

### 2.1 Drug Extraction

Two hundred and fifty dried LBP (procured from Hubei Jikang Pharmaceutical Co., Ltd.) were soaked in 1250 ml of water for 30 minutes. Then, a heat reflux extraction was performed until it reached a micro-boiling state, and it was maintained for 1 hour. The mixture was filtered, and the extraction process was repeated with an additional 1250 ml of water, followed by filtering again. The extracts from both rounds were combined. The resulting dry powder extract was frozen in a -80°C refrigerator and subjected to vacuum drying in a freeze dryer. Finally, the extract was sealed and stored at room temperature.

### 2.2 Experimental Animal

Specific-pathogen-free male Balb/c mice of 8-week-old, body weight 20 ± 2 g, were purchased from Beijing Experimental Animal Research Center (Production License Number: SCXK 2016-0006). The animals were divided into 3 groups, *i.e*., 1) the normal control group (N-C) of 10 mice without irradiation, but with oral saline administration; 2) the experimental control group (Exp-C) of 10 mice with X-ray total body irradiation at 5.5 Gy and oral saline administration; 3) the LBP-treated group (Exp-LBP) of 10 mice with X-ray irradiation at 5.5 Gy and LBP extract administered orally at 10 g / kg per day 2 h after irradiation, continued for 4 weeks. Four weeks after irradiation, the behavioral tests, including the open field test and water maze test, were conducted. The body weight was recorded before, as well as in the first, second, third, and fourth weeks after irradiation.

### 2.3 Open Field Experiment

The SuperMaze animal behavior record and analysis system for open field test (Shanghai Xinran Mdt InfoTech Ltd) was utilized for the open field experiment. The field size of 50 cm (length) × 50 cm (width) × 30 cm (height) was divided into 16 boxes. The total movement distance covered and the time spent in the central area (central 4 boxes) within 5 5-minute test period were recorded.

### 2.4 Water Maze Test

The standard Morris water maze program was implemented in reference to our previous study [29, 30]. In brief, mice were trained for 6 consecutive days to search for hidden platforms in a water maze, and four quadrants were conducted daily with an interval of 30 seconds. They were only allowed to search on the platform for less than 60 seconds. If a mouse could not find the platform, it was guided to the platform and remained there for 30 seconds. In each experiment, the Nordus video tracking system (Ethovision, Nordus Information Technology, Holland) was used to record the swimming path and locate the latency of the hidden platform. Every day, the mice were released from the first quadrant, the duration from the start of release to finding the central platform was recorded and calculated as the escape time. In this study, learning ability was quantified as the escape time on day 6. The shorter the escape time required for a mouse to find the central platform on the 6th day, the higher its spatial memory score.

### 2.5 The Levels of GPx and MDA Detected by ELISA

Mouse blood samples were collected and allowed to stand at room temperature for 40 minutes to 1 hour (no more than 1 hour). The samples were placed on ice for processing (no more than 4 hours), or they were centrifuged in a timely manner. The blood was analyzed and subjected to bleeding clearance and then centrifuged at 3000 rpm (1000 g) at 4°C for 10 minutes. The supernatant was collected and used to detect GPx and MDA expression by enzyme-linked immunosorbent assay (ELISA). All detection kits were purchased from Abcam, England (refer to the kit manual for the specific protocol). A standard curve was generated, and the concentration of each test sample was calculated.

### 2.6 Immunohistochemistry

Six weeks after irradiation, the mice were anesthetized with 1% pentobarbital, transcardially perfused with saline to wash out the blood and fixed with 4% paraformaldehyde. The brain was removed, postfixed and transferred to 30% sucrose solution in 0.1 M phosphate buffer (pH: 7.4) for preservation. Sagittal sections were then cut to a thickness of 50 μm. A set of 3 serial sections was prepared and placed individually in different wells of a 24-well tissue culture dish for further Doublecortin (DCX) and parvalbumin (PV) immunohistochemical staining. For immunostaining, free-floating sections were first treated with 3% H_2_O_2_ and blocked in 2% donkey (for DCX) or goat (for PV) serum. Two hours after blocking, sections were incubated with goat anti-DCX (Santa Cruz Biotechnology Inc., CA, USA), and rabbit anti-PV (Swant, Switzerland) at 4°C overnight. The sections were then treated with donkey anti-goat (for DCX) or goat anti-rabbit (for PV) second antibodies for 1 h. After incubation in AB solution (ABC Kit, Vector Laboratories Inc., Burlingame, CA, USA), they were treated in DAB horseradish peroxidase chromogenic reagent for 10 min (Vector Laboratories Inc., Burlingame, CA, USA). Sections were then mounted on slides, dried naturally, and then coverslipped.

### 2.7 Statistical Analysis

The total cells labeled with DCX or PV in the hilus of the dentate gyrus in the dorsal hippocampus were counted using stereological methods using STEREOLOGERTM software (Stereological Resource Center Biosciences, Inc. Florida, USA). The data were analyzed using GraphPad and Prism 6 and expressed as mean ± standard deviation (‾x ± s). Data from various groups were compared using one-way ANOVA and Student t-test. *p* < 0.05 was considered statistically significant.

## 3 RESULTS

### 3.1 Effects of LBP Extract on the General Condition and Body Weight of Radiation-exposed Mice

Fig. (**1**) revealed that compared with the normal group, the radiation group mice showed significant weight loss accompanied by decreased activity and decreased diet (*p* < 0.01). The LBP treatment group significantly improved these parameters (*p* < 0.01). These findings indicate that ionizing radiation significantly prevents weight gain in mice. However, the administration of LBP alleviates this weight gain inhibition induced by ionizing radiation.

### 3.2 Open Field Experiment

**Fig. (2):**
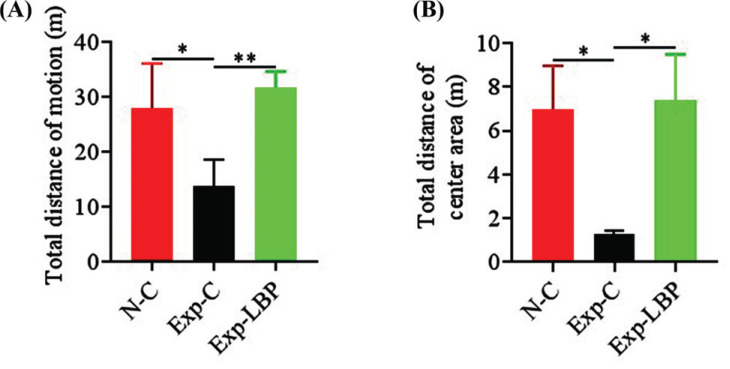
Effect of LBP extract on the emotional state of radiation-injured mice. (**A**) Total distance covered in the open field movement; (**B**) Total distance covered in the central area of the open field. (*p < 0.05; **p < 0.01).

In the open-field experiment, the total distance covered in the open-field area was significantly longer in the normal group than in the irradiated group (*p* < 0.05) (Fig. **2A**). Moreover, the total distance covered in the open field area was also significantly longer in the LBP group (*p* < 0.01). In the central area of the open field, the total distance covered by mice in the LBP group was significantly longer relative to that of the irradiated group (*p* < 0.05) (Fig. **2B**). These results showed that X-ray exposure induced depression in mice, and LBP extract improved depression levels.

### 3.3 Water Maze Test

**Fig. (3):**
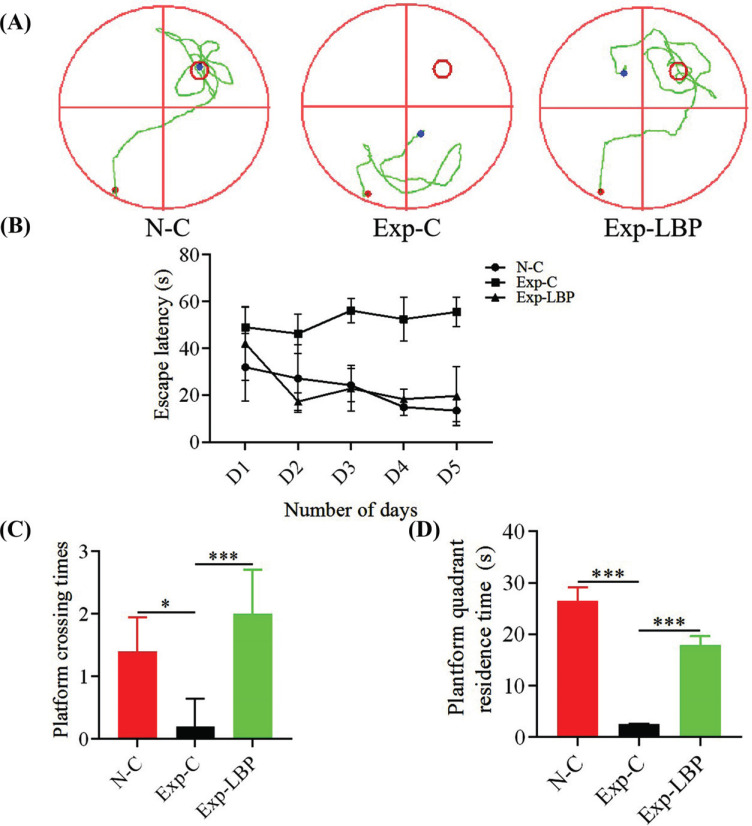
Effects of LBP on the learning and memory of radiation-injured mice. (**A**) The swimming trajectory map of exploration opposite platform in the water maze test; (**B**) Water maze escape incubation period; Exp-C *vs*. N-C, *p* < 0.01; Exp-C *vs*. Exp-LBP, *p* < 0.01. (**C**) Number of across the platform in the water maze test; (**D**) The exploration time of the quadrant that the platform located in the water maze test (*p<0.05; ***p<0.001).

In the first 5 days, mice were placed in water in the first, second, third and fourth quadrants, and trained four times a day. On the sixth day, the platform was removed and mice were placed in the contralateral quadrant of the original platform. They were then allowed to search for the original platform under the guidance of visual cues. Compared with the normal group and LBP treatment group, the escape latency of irradiated mice was significantly longer during the training experiment on the 4th to 5th day (*p* < 0.01, Figs. **3A** and **B**). The time spent by irradiated mice in the platform quadrant and the number of times animals crossed the platform were significantly reduced (*p* < 0.05, *p* < 0.001, Figs. **3C** and **D**). These findings indicate that ionizing radiation does indeed impair the cognitive behavior, learning, and memory abilities of mice. However, administration of LBP extract significantly ameliorated these declines in cognitive behavior and learning and memory abilities in mice.

### 3.4 ELISA Experiment

Data shown in Fig. (**4A**) showed that the serum GPx content of the irradiated group was significantly decreased compared with the normal group (*p* < 0.05). Compared with the irradiated group, the serum GPx content of the LBP group was significantly increased (*p* < 0.05). As shown in Fig. (**4B**), compared with the normal group, the serum MDA content of the irradiated group was significantly increased (*p* < 0.01), compared with the irradiated group, the serum MDA content of the LBP group was significantly decreased (*p* < 0.001). The results indicated that the LBP extract suppressed the oxidative stress network and protected mice against irradiated-induced damage.

### 3.5 Immunohistochemistry

As shown in Figs. (**5A** and **B**), there was a significant reduction in the number of DCX-positive cells in the irradiated group compared to the normal group (*p* < 0.001). However, after treatment with LBP, the number of DCX-positive cells in the LBP group showed a significant increase (*p* < 0.01). These outcomes indicate that the administration of LBP extract has the potential to counteract the decrease in DCX, which is a characteristic protein of neuronal precursor cells following radiation-induced injury. Figs. (**5C** and **D**) shows that, compared with the normal group, the number of PV-positive cells in the irradiated group was significantly decreased (*p* < 0.05). After LBP treatment, the number of PV-positive cells in the LBP group was significantly increased (*p* < 0.001). The results indicated that exposure to 5.5 Gy radiation led to the loss of PV number of interneurons in the dentate gyrus region of the brain hippocampus. Administration of LBP extract alleviated this effect and reduced the loss of neuron number.

## 4 DISCUSSION

### 4.1 LBP Can Improve the Overall State and Learning and Memory Capacity of Mice Exposed to Irradiation

Radiation may cause systemic damage, resulting in weight loss, loss of appetite and deterioration of state. Studies have shown that the weight of 5-week-old rats exposed to low-dose X-ray irradiation was significantly lower compared with that of non-irradiated rats about 10 weeks after irradiation, and the weight difference lasted until 30 weeks after irradiation [31, 32]. Here, we obtained consistent results following exposure to radiation. Particularly, mice exhibited significant weight loss, and regained the weight three weeks later. Interestingly, LBP led to a temporary increase in the weight of mice. This effect was attributed to its ability to promote the body's self-healing following irradiation-induced injury.

The most common side effects of radiation therapy are radiation-induced cognitive impairment and emotional disorders. Inhibition of neurogenesis caused by radiation-induced neural stem cell damage has been shown to be an important mechanism mediating radiation-induced cognitive impairment. The hippocampus plays a key role in learning and memory [33-35]. Yang *et al*. injected exosomes derived from GL261 cells into the hippocampus of mice. They found that this significantly inhibited neurogenesis and cognitive impairment after two months, whereas exosomes derived from irradiated GL261 cells induced even greater inhibition [36]. After exposure to 6 Gy γ-ray, the length of stay in the open-field experimental central area of mice was decreased suggesting that the irradiation caused depression in mice [37-39]. Another study reported that pretreatment with Lycium barbarum polysaccharides significantly improved learning and memory in a rat model of scopolamine-induced dementia as indicated by a novel object/object location recognition task and a Morris water maze test [40]. In our study, mice exposed to radiation found it difficult to find the area where the platform was located in the Morris Water maze experiment, indicating that the radiation impaired neurogenesis in the hippocampus of mice thereby weakening the learning and memory ability of mice. In the open-field experiment, the residence time of mice exposed to radiation in the central area of the open field was reduced suggesting that radiation-induced depression in mice. Following the administration of LBP, the learning and memory capabilities, as well as the depressive tendencies of mice were significantly improved.

### 4.2 Antioxidant Mechanism of LBP Extract

Radiation exposure can trigger the rapid production of ROS, as a result of the ionization of water molecules and the direct ionization of target molecules as well as the generation of free radicals and reactive oxygen species [41-43]. In addition to the rapid burst of free radicals and ROS observed immediately, cells exhibit a sustained and long-term increase in ROS from a few minutes to a few days after irradiation. The central nervous system is inherently susceptible to oxidative stress because the central nervous system has a highly active oxidative metabolism, which generates relatively high levels of intracellular ROS production [44-46]. Radiation can also induce endogenous ROS production in mitochondria and alter the permeability of the mitochondrial membrane, which allows the production of ROS. Endogenous antioxidant systems protect against radiation-induced oxidative stress by scavenging free radicals. Lycium barbarum and polychlorin have been found to be effective antioxidants in numerous studies. For example, Li *et al.* demonstrated that in an aged mouse model, oral administration of Lycium barbarum polysaccharide effectively improved the level of antioxidant enzymes GPx and catalase, and reduced the formation of MDA, a key final product of oxidative stress [47-49]. LBP increased the number of newly formed neurons in the dentate gyrus of the hippocampus and attenuated abnormal oxidative stress by restoring levels of antioxidant enzymes such as GPx [50]. Huangjing significantly reduced the content of MDA in skeletal muscle and serum, reduced the activity of free radicals, and increased the activity of GPx [51, 52]. A study by Huang *et al.* showed that Huangjing prevented depression-like behaviors and synaptic and neuronal damage by reducing ROS and inflammatory responses [53]. In our study, the administration of LBP extract increased the content of GPx and decreased the content of MDA in serum, which indicated that the LBP extract exerted significant improvement in radiation-induced oxidative damage.

### 4.3 LBP Extract Prevents Radiation-induced Loss of Hippocampal Interneurons

Doublecortin (DCX) is a microtubule-associated protein that participates in the neurodevelopment of neurons during migration and differentiation [54-56]. DCX immunostaining provided accurate quantification of the absolute number of new neurons generated and dendritic growth in the dentate gyrus of the adult hippocampus over a 12-day period. This is particularly useful and it allows the analysis of changes in dentate neurogenesis during the progression of neurodegenerative diseases in the human hippocampus [55, 57-59]. This study revealed that low-dose radiation reduced hippocampal dentate gyrus neurogenesis and disrupted hippocampal function which increased the risk of cognitive impairment. LBP treatment promotes the regeneration of neurons.

The activity of parvalbumin (PV)-positive interneurons in the dentate gyrus is a crucial factor influencing cognitive function [60-63]. Changes in cognitive function caused by radiation exposure are thought to be due to the loss of hippocampal-dependent learning and memory functions, including spatial information processing. This is believed to be associated with alterations in neuronal progenitors located in the subgranular zone of the hippocampus. The cognitive function of the brain is intricately linked to the structure and functioning of pyramidal cells and fast-spiking interneurons that express small albumin [64-67]. Several studies have demonstrated that ionizing radiation affects GABAergic neurotransmission, including in PV neurons [68-72]. This effect is strongly linked to oxidative stress generated by ionizing radiation, with PV neurons being the major producers of reactive oxygen species, accounting for 50% of gabaergic interneurons [73]. In the pilocarpine-induced status epilepticus mouse model, PV immunopositive neurogenesis was significantly reduced in different regions of the hippocampus, suggesting that these biomarkers may prevent neuronal loss and neuronal regeneration in the hippocampus [74]. In this study, 5.5 Gy radiation exposure caused the loss of DCX and PV immunopositive cells in the dentate gyrus region of the hippocampus, and LBP extract could effectively prevent the loss of these two cells.

## CONCLUSION

A 5.5 Gy dose of ionizing radiation effectively induces depressive behavior and spatial memory impairment in BALB/c mice. This observation has been corroborated by our patented invention in China (ZL 2018 1 0366371.X). Oral LBP prevented the loss of hippocampal neurons and improved radiation-induced cognitive impairment and depression. Although we have demonstrated that LBP can regulate radiation-induced oxidative stress, there is a need for further investigations to elucidate the molecular mechanism of LBP. Research has strongly suggested that this traditional Chinese Medicine prescription may be a promising neuroprotective supplement against radiation-induced injury that can prevent cognitive impairment induced by radiation therapy or other ionizing radiation. Given its high efficiency and low toxicity, LPB can be used to develop new anti-radiation drugs.

## Figures and Tables

**Fig. (1):**
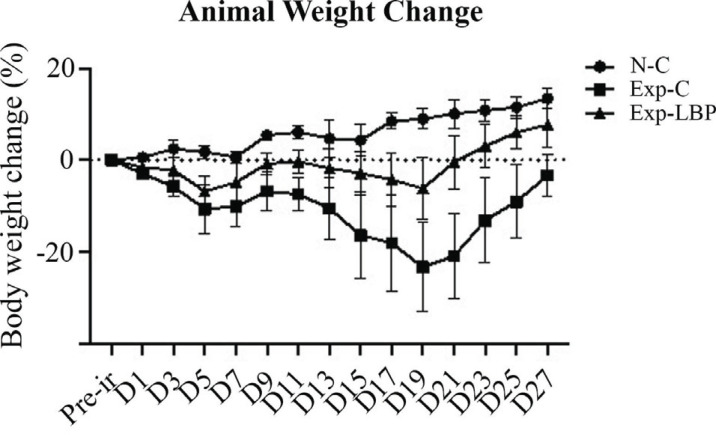
Changes in weight change of mice after 5.5 Gy X-ray irradiation for four weeks. Exp-C *vs*. N-C, *p* < 0.01; Exp-C *vs*. Exp-LBP, *p* < 0.01.

**Fig. (4):**
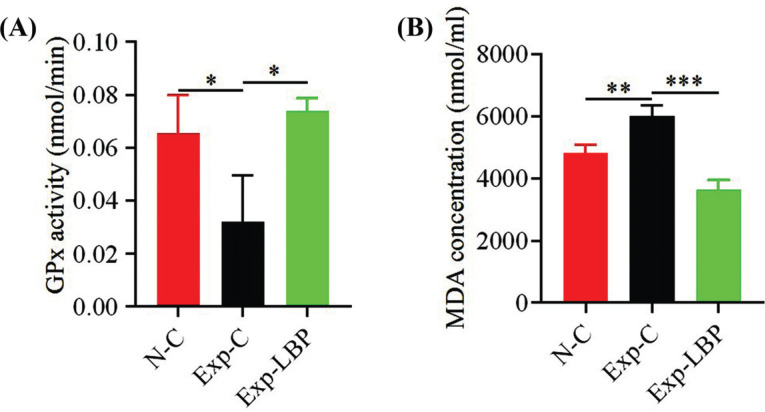
Measurement of serum GPx and MDA concentrations by ELISA. (**A**) Changes in serum enzyme GPx content per minute following treatment with LBP extract in 5.5 Gy irradiated mice; (**B**) Effect of LBP extract on serum MDA content from radiation-damaged mice (*p < 0.05; **p < 0.01; ***p < 0.001).

**Fig. (5):**
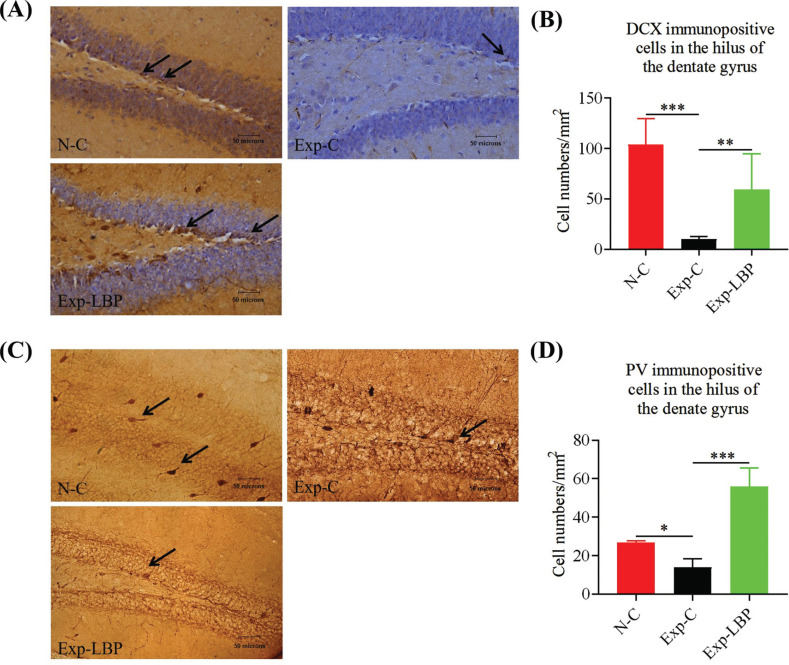
Quantitative analysis of DCX-positive cells in mouse hippocampal dentate gyrus by immunohistochemistry. (**A**) Immunohistochemical-stained sections for DCX; (**B**) Number of DCX-positive expression in dentate gyrus of hippocampus; (**C**) Immunohistochemical-stained sections for PV; (**D**) Number of PV-positive expression in dentate gyrus of hippocampus. (*P < 0.05; **P < 0.01; ***P < 0.001).

## Data Availability

The data and supportive information are available within the article.

## LIST OF ABBREVIATIONS

LBP: Lycium Barbarum Pills
IR: Ionizing Radiation
ROS: Reactive Oxygen Species
MDA: Malondialdehyde
ELISA: Enzyme-Linked Immunosorbent Assay
PV: Parvalbumin
